# Filamentary Resistive Switching Mechanism in CuO Thin Film-Based Memristor

**DOI:** 10.3390/ma18163820

**Published:** 2025-08-14

**Authors:** Monika Ozga, Robert Mroczynski, Krzysztof Matus, Sebastian Arabasz, Bartłomiej S. Witkowski

**Affiliations:** 1 Institute of Physics of the Polish Academy of Sciences, Al. Lotnikow 32/46, 02-668 Warsaw, Poland; bwitkow@ifpan.edu.pl; 2 Warsaw University of Technology, Institute of Microelectronics and Optoelectronics, Koszykowa 75, 00-662 Warsaw, Poland; robert.mroczynski@pw.edu.pl; 3 Materials Research Laboratory, Faculty of Mechanical Engineering, Silesian University of Technology, Konarskiego 18A, 44-100 Gliwice, Poland; krzysztof.matus@polsl.pl; 4 Lukasiewicz Research Network-PORT Polish Center for Technology Development, 147 Stablowicka, 54-066 Wroclaw, Poland; sebastian.arabasz@port.lukasiewicz.gov.pl

**Keywords:** CuO, conductive filaments, hydrothermal method, memristor, resistive switching

## Abstract

Understanding the resistive switching (RS) mechanisms in memristive devices is crucial for developing non-volatile memory technologies. Here, we investigate the memristor effect in hydrothermally grown Au-nanoseeded CuO films. Based on I-V measurements, conductive-AFM, S/TEM, and EDS analyses, we examine the changes within the switching layer associated with RS. Our results reveal a filamentary mechanism of RS. Notably, EDS mapping shows directional Au redistribution between the bottom nanoseeds and the top electrode, while Cu and O remain uniformly distributed. These findings support an electrochemical metallization (ECM)-like filamentary mechanism driven by Au species migration. The use of Au-nanoseeds, required by the solution-based growth method, critically affects filament formation and RS behavior. Our results emphasize the importance of microstructure and electrode–oxide interfaces in determining the switching mechanism in oxide-based memristors.

## 1. Introduction

Today, a memristor is commonly used to describe a broad class of two-terminal devices that exhibit resistive switching (RS) phenomena. While many implementations do not fully align with Chua’s original definition from 1971 [1], the terms memristor and resistive switching device, as well as memristor effect and resistive switching phenomenon, are often used interchangeably. Chua himself acknowledged this broader interpretation, stating that any two-terminal electrical device that exhibits the property of switching between two resistance states under the influence of an appropriate electrical pulse and whose state can be read in low electric field conditions can be considered a memristor [2].

Memristive devices have been shown to undergo many (above 10^8^) reversible switching cycles via electrical pulses [3]. Many memristive devices exhibit non-volatile memory behavior, retaining their programmed resistance state after the removal of the electrical power or programming pulse [4]. Due to their advantages, such as low power consumption, high scalability, fast switching speeds, and the ability to mimic synaptic behavior, memristive devices are considered promising candidates for emerging technologies, including next-generation non-volatile memory technologies [5,6], neural networks [7], artificial intelligence [8], and neuromorphic computing [9]. Following the first demonstration of the memristor by HP Labs, interest in this area increased rapidly. Current research efforts range from developing switching materials and optimizing fabrication processes to designing advanced device architectures and in-depth studies of the physical mechanisms underlying resistive switching and memory effect. This comprehensive approach aims not only to enhance device performance and scalability but also to ensure reliability and reproducibility in practical applications.

Resistive switching refers to a phenomenon in which a material’s electrical resistance changes between high and low resistance states (HRS and LRS, respectively) because of physical processes occurring under the influence of an external electric field. Two primary mechanisms of RS are identified in the literature. The first involves charge trapping and de-trapping at the interface between the active layer and the electrode, leading to redistribution of carriers and modification of the Schottky barrier, resulting in resistance changes [10,11]. The second mechanism attributes RS to the formation and rupture of conduction filaments (CF) within the switching layer. These filaments, composed of metal atoms, metal ions, or oxygen vacancies, are formed through the electric field-driven migration of cations or anions under an applied voltage [10].

The resistive switching phenomenon has been observed in a wide range of materials, including a large group of metal oxides. Among them, the most extensively studied oxides are HfO_x_, AlO_x_, NiO_x_, TiO_x_, and TaO_x_ [12,13,14,15,16]_._ Cupric oxide remains a less commonly explored material in this field, despite its advantageous properties, including CMOS compatibility, low toxicity, and abundance. Most RS studies involving CuO have focused on heterostructures, such as GaIn/TiO2-CuO/ITO [17] and Cu/CuO/ZnO/AZO [18] or compositionally graded (Ti–Cu)O_x_-based structures [19]. In comparison, reports on single-layer CuO-based resistive random access memory (RRAM) structures are rarely reported. Notable examples include structures where CuO was deposited using the sol-gel method -Ag/CuO/n^+^-Si [20] or magnetron RF sputtering Al/CuO/ITO [21], Ag/CuO/ITO [22], and Pt/CuO_x_/Pt [23]. Hydrothermal synthesis has also been used to fabricate Al/CuO/stainless steel devices [24], although that study focused primarily on synaptic functionality rather than the resistive memory performance. Given the current state of research, our structures -Al/Si (n^+++^)/Au-nanoseeds/CuO/Au represent a unique solution in terms of both the single-phase active layer structure and the utilized fabrication method [25] demonstrating both technical innovation and practical relevance, as confirmed by the granted intellectual property rights detailed in the Patents Section 6.

In the present study, we employ a complementary set of techniques, including current-voltage (I–V) measurements, conductive atomic force microscopy (C-AFM), transmission electron microscopy (TEM), and energy-dispersive X-ray spectroscopy (EDS), to identify the resistive switching mechanism of single-phase CuO-based memristive devices. This multi-technique approach confirmed the filamentary switching mechanism and allowed the identification of the chemical composition of conductive filaments.

## 2. Materials and Methods

### 2.1. Single-Layer CuO Memristor Structure

**Figure 1:**
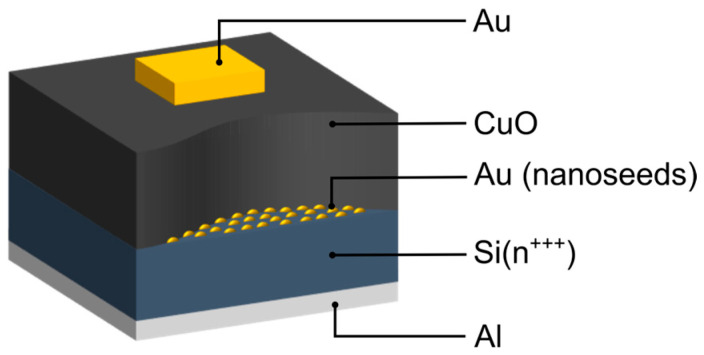
Graphical representation of the examined structures.

The resistive switching phenomenon was investigated using metal–insulator–metal (MIM) structures in which copper (II) oxide served as an insulating layer. The CuO thin films (with a thickness in the range of 50−100 nm) were hydrothermally synthesized on a properly prepared, highly doped silicon substrate (500 μm) exhibiting metallic-like conductivity. In these devices, aluminum (100 nm) was used as the bottom electrode, while the top-point electrodes were deposited from gold (100 nm for microscale electrical measurements and 15 nm for TEM lamellae). Aluminum contacts were deposited using a PlasmaLab Oxford System 400 magnetron sputtering (Oxford Instruments, Bristol, UK), and gold contacts of rectangular shape (150 nm × 250 nm) were fabricated using a Quorum Q150T ES coater (Quorum Technologies Ltd., Laughton, UK). For experiments involving current mapping via the conductive atomic force microscopy technique, the top Au contact was replaced with a conductive PtIr-coated AFM probe, which simultaneously served as the top electrode and scanning element. The structure investigated in this work is schematically depicted in Figure 1.

#### 2.1.1. Substrate Preparation

CuO films were synthesized on highly conductive n-type silicon substrates (R = 0.001 Ω·cm), which underwent a multi-step cleaning and surface preparation procedure. First, the native silicon oxide (SiO_2_) layer was removed by immersing the substrates in a 40% hydrofluoric acid (HF) for one minute. Subsequently, a modified RCA cleaning protocol was applied, including subsequent treatments with Piranha solution, standard clean-1 (SC-1), standard clean-2 (SC-2), and a final HF dip. Each cleaning step was followed by rinsing the substrates three times with deionized water to remove residual chemicals.

**Figure 2:**
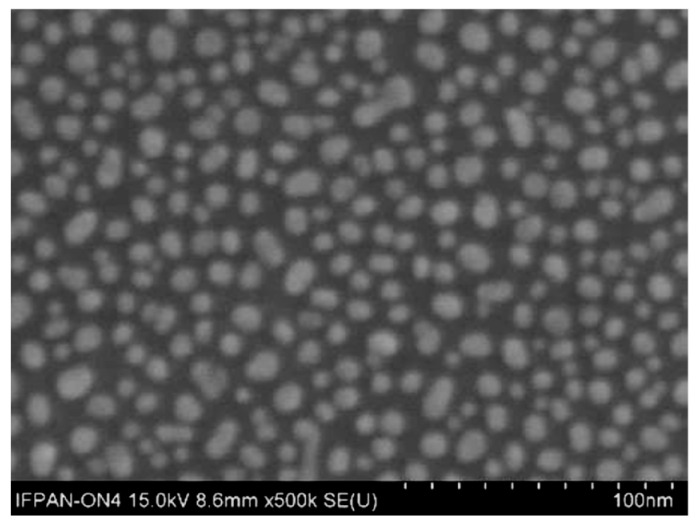
SEM image of the substrate covered with Au-nanoseeds.

**Figure A1:**
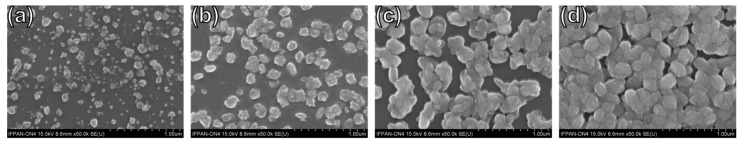
SEM images showing the growth of the CuO layer on gold nanoseeds, shown in Figure 2: (**a**) after 30 s, (**b**) after 60 s, (**c**) after 90 s, and (**d**) after 120 s of the hydrothermal growth process.

Controlled substrate nucleation is a critical step to induce the growth of nanostructures on the surface, which would otherwise occur exclusively as nanopowder formation within the volume of the reaction mixture. To facilitate nucleation, a 1.5 nm gold layer was deposited onto the so-prepared substrate using a turbomolecular-pumped coater (Quorum Technologies Ltd., Laughton, UK). During the deposition process, the substrate is placed on a rotating stage and is not externally heated. The total deposition time is 1 min, including 40 s allocated for target pre-cleaning. Although a slight increase in substrate temperature may occur due to plasma exposure, it does not exceed 50 °C. Upon deposition, this thin gold film spontaneously reorganizes into nanoislands, as illustrated in Figure 2. This behavior is consistent with literature reports on the morphological evolution of discontinuous metal films at the nanometer scale [26]. Ensuring proper nucleation is critical for directing the growth of the CuO layer onto the substrate surface, rather than only within the volume of the reaction mixture. As shown in the Appendix (Figure A1), SEM images of sample surfaces extracted from the reactor at various times of the growth process demonstrate that the gold nanoseeds serve as nucleation sites for CuO growth on the substrate. Subsequently, CuO grows laterally and vertically, following the mechanism described in [27].

Gold has been used by other research groups [28,29] to promote the formation of various materials from aqueous solutions. However, prior to our first report introducing this growth technique [27], no literature reports demonstrated the use of gold as a nucleating agent specifically for CuO films. It highlights the novelty and significance of our approach.

#### 2.1.2. Synthesis of CuO Thin Films

CuO thin films were grown via an innovative approach to the hydrothermal method with ultra-fast growth rate and mild synthesis conditions, specifically, low reaction temperature (below 100 °C) and atmospheric pressure [27]. This eco-friendly and cost-effective technology avoids the use of toxic or expensive reagents and does not require sophisticated equipment. To enhance the thermal and electrical stability of as-grown films, a cyclic procedure combining hydrothermal synthesis with rapid thermal annealing (HT + RTA) was applied, following the procedure described in detail in [30].

First, the hydrothermal growth process was carried out. For this purpose, a 1 mM aqueous solution of copper (II) acetate (99.9%, Chempur) was prepared, whose pH was adjusted to 6.5 by adding a 1M NaOH (Stanlab) solution. The resulting mixture, along with the appropriately prepared substrate, was placed in an open reaction vessel externally heated on an induction plate (Stalgast, Warsaw, Poland) using a power of 3.5 kW. After reaching boiling temperature, the reaction continued for an additional 30 s before the heating source was turned off. The substrates were then removed, rinsed in an ultrasonic bath with isopropanol for 1 min, and dried under a nitrogen stream. Subsequently, rapid thermal annealing (RTA, AccuThermo AW610, Allwin21, Morgan Hill, CA, USA) was performed at 450 °C in a mixed O_2_/N_2_ atmosphere (1:1) for 5 min. This HT + RTA sequence was then repeated a second time on the same substrate, resulting in two full synthesis–annealing cycles.

### 2.2. Measurement Equipment

#### 2.2.1. Electron Microscopy Techniques

The CuO surface morphology and cross-sectional imaging of the structures were analyzed using a scanning electron microscope (SEM, SU-70, Hitachi, Tokyo, Japan). Transmission electron microscopy (TEM) observations were performed using a S/TEM TITAN 80-300 (FEI, Hillsboro, OR, USA) microscope operating at an accelerating voltage of 300 kV. The instrument was equipped with an energy-dispersive X-ray spectroscopy (EDS) system (EDAX, Mahwah, NJ, USA). To enable cross-sectional imaging and high-resolution structural analysis of the studied structures, electron beam-transparent lamellae were prepared directly from the active device region using a DualBeam Focused Ion Beam–Scanning Electron Microscope (FIB-SEM) system (Helios 450HP, Thermo Fisher Scientific, Waltham, MA, USA) equipped with an LMIS Ga^+^ source.

#### 2.2.2. Scanning Probe Microscopy Techniques

The surface topography of the CuO films and local nanoelectrical measurements were carried out using a scanning probe microscope (SPM, Dimension Icon, Bruker, Santa Barbara, CA, USA). Topography imaging was performed using atomic force microscopy (AFM) in peak force tapping mode with a ScanAsyst-AIR probe. To enable electrical characterization, the system was equipped with a TUNA2 module capable of detecting currents in the range of 80 fA to ~1μA. Conductive atomic force microscopy (C-AFM) investigations were carried out in contact mode using SCM-PIT-V2 probes with PtIr conductive coating. Both the probes and the module were supplied by Bruker.

#### 2.2.3. I–V Measurements

Microscale electrical characterization was performed using a Keysight B1500A semiconductor characterization system (Keysight Technologies, Santa Rosa, CA, USA) using a SUSS PM-8 probe station equipped with a probe–shield system. All measurements were conducted at room temperature (25 °C) under ambient conditions.

#### 2.2.4. Data Processing and Software

The acquired data were processed and visualized using OriginPro 2023, ImageJ (ver. 1.54 g), and Nanoscope Analysis (ver. 3.00).

## 3. Results

### 3.1. Evidence of Resistive Switching and Memristor Performance

The resistive switching behavior of the CuO-based memristor structure was previously confirmed under ambient conditions. The device demonstrated bipolar resistive switching and stable high and low resistance states [25]. To provide spatially resolved confirmation of memristive functionality, C-AFM was employed following a previously validated procedure [31]. To make the mapping possible, measurements were performed directly on the CuO layer and not on the electrical contact (Au). The force setpoint was kept constant during all scans to ensure repeatable electrical contact conditions.

Before the current mapping, several I–V curves were recorded at randomly selected positions on the sample using the spectroscopic mode of the microscope. These measurements determined the switching voltages-V_set_ and V_reset_, required to switch the structure between individual resistance states. The most reproducible switching was observed for ± 8 V.

Based on these parameters, localized RS was induced by scanning designated sample regions under controlled bias applied to the bottom electrode from the microscope stage. First, a 3 × 3 μm^2^ area was set to the low resistance state (LRS) by applying V_set_ = + 8 V. Subsequently, a smaller 1 × 1 μm^2^ area within this region was reset to a high resistance state (HRS) by applying V_reset_ = −8 V during scanning. The final state of the device was read out after ~30 min by acquiring a current map over a 5 × 5 µm^2^ area at a constant read voltage V_read_ = 1 V.

**Figure 3:**
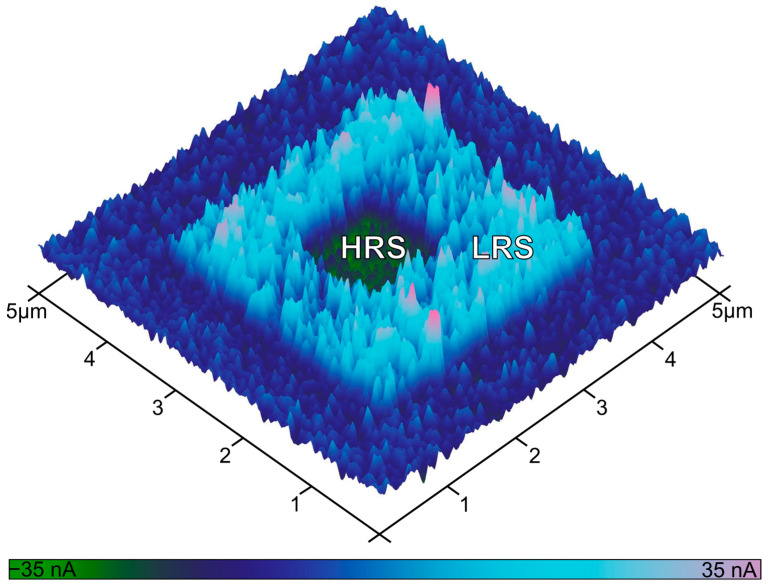
C-AFM current map acquired for V_read_ = 1 V showing three distinct regions: unwritten (dark blue), LRS (light blue), and HRS (green).

The resulting current image (Figure 3) distinctly reveals three areas corresponding to the unwritten (dark blue), the LRS-switched (light blue), and the reset HRS (green) regions. Quantitative analysis of the recorded current revealed root mean square current values of 14 nA for the LRS, 5.4 nA for HRS, and 6.6 nA for the unwritten area. These results provide direct nanoscale-resolved evidence of non-volatile resistive switching in the CuO-based structure.

Following the microscale room temperature investigation of RS [25], further characterization was performed by placing the probe on the top Au contact (150nm × 250nm) at an elevated temperature of 100 °C using a probe station. This temperature slightly exceeds the typical upper limit for standard electronic operation (85 °C) and was intentionally selected to introduce a controlled thermal margin, allowing evaluation of memristor functionality under more demanding, application-relevant conditions.

**Figure 4:**
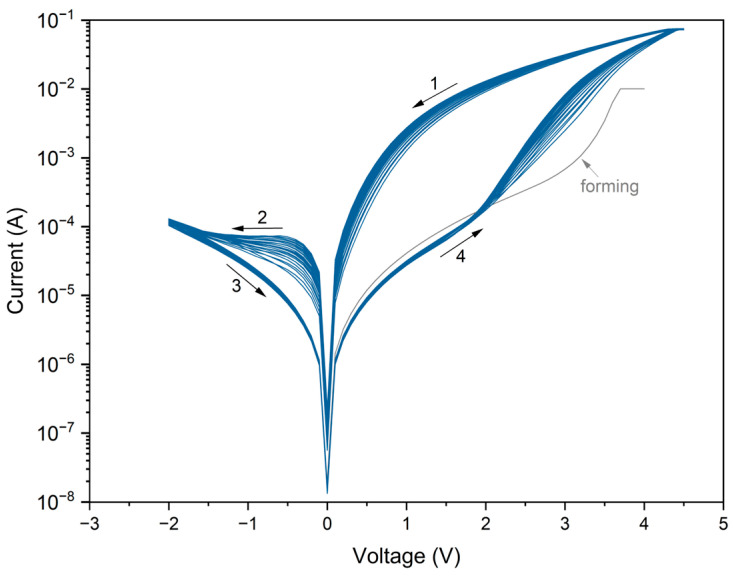
Representative I–V characteristics of a CuO-based memristor over 25 cycles at 100.

The device was first subjected to a forming process. A voltage sweep from 0 to +4 V was applied with a compliance current of 0.01 A. The forming curve does not exhibit typical dielectric breakdown. Instead, the current increases gradually and reaches the LRS without an abrupt transition, with a noticeably steeper rise above +3 V. Figure 4 shows twenty-five consecutive I–V cycles acquired in a voltage sweep: from +4.5 V to ±2 V to +4.5 V, with a compliance current of 0.075 A. The resulting curves exhibit a characteristic pinched at 0 V hysteresis loop, confirming bipolar memristive behavior.

At a V_read_ = +1 V, the LRS/HRS current ratio reaches approximately two magnitude orders. In contrast, significantly lower LRS/HRS ratios are observed in negative polarization. At V_read_ = −1 V, the ratio is ~2, while at V_read_ = −0.5 V it reaches ~10. One order of magnitude is the value often regarded as the minimum acceptable threshold for practical memory applications.

Although some cycle-to-cycle variations in I–V curves are evident across the initial switching loops, the response becomes progressively more stable in subsequent sweeps. This trend is likely associated with the sample’s thermal equilibration. Since the temperature was monitored at the level of the heating stage rather than directly on the device, we attribute this behavior to the slight gradual warming of the sample to its target operational temperature. Once thermal stabilization was achieved, the I-V curves exhibited improved overlap and reproducibility. It should be noted that the influence of elevated temperature on the switching dynamics and conduction mechanisms is a complex issue, but it is beyond this study’s scope. Further investigations are planned to explore this aspect in detail.

### 3.2. Conduction Mechanism

**Figure 5:**
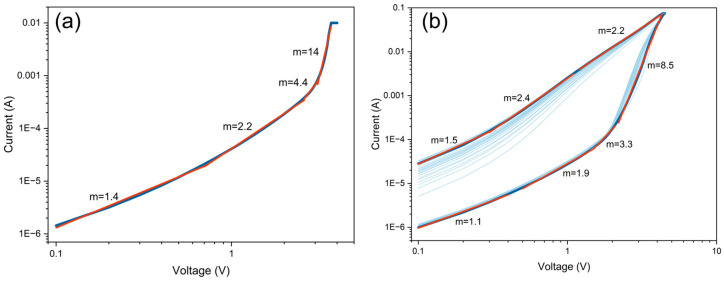
Double-logarithmic representation of the (**a**) forming and (**b**) switching I–V curves (extracted from the positive voltage range), including linear fits and corresponding slope values (m).

Preliminary studies conducted at room temperature indicated that the dominant conduction mechanism in the investigated memristive structures follows the space charge limited current (SCLC) model [32]. This mechanism is widely used to describe charge transport in resistive switching devices, particularly those based on the formation and rupture of CF [33]. When the I-V characteristics are plotted on a double-logarithmic scale, the SCLC model typically reveals three distinct conduction regimes described by the slope $m=\frac{dlog(I)}{dlog(V)}$ whereby $I\propto V^{m}$. These include an initial Ohmic region (m = 1) followed by trap-limited SCLC (m = 2), in which injected carriers are progressively captured by the traps until, at critical voltage, all present traps become filled. Beyond this point, in the trap-free regime (m > 2), the current increases rapidly as injected carriers dominate the current flow after trap saturation [34,35]. In this work, we extend this analysis to I-V characteristics recorded at elevated temperature (100 °C). The following analysis is focused on electrical conduction behavior, while the nature and chemical composition of the conductive filaments are examined in subsequent sections. Figure 5 shows the corresponding log-log plots for the positive voltage sweep, including both the forming trace and representative switching cycles.

The forming curve plotted on a double-logarithmic scale (Figure 5a) reveals a sequence of distinct conduction regimes characterized by slopes of 1.4, 2.2, 4.4, and 14. The initial three regions closely follow the SCLC model assumptions mentioned above. Notably, an additional fourth regime is observed, exhibiting an exceptionally steep slope of 14. Such a rapid increase in current is likely associated with the transition from classical SCLC behavior to filamentary conduction [36]. This abrupt transition occurs when the electric field and current density reach critical thresholds, rapidly forming highly conductive filaments within the switching layer.

A similar progression is observed for the cycle-to-cycle HRS-to-LRS switching (set operation) curves shown in Figure 5b. When the device is in HRS, the current evolves through regimes with slopes of 1.1, 1.9, 3.3, and 8.5. Once again, the final steep slope supports the interpretation of CF formation under a high electric field. Following abrupt set operations, the device switches into LRS, where the current is characterized by slopes of 2.2, 2.4, and 1.5. This suggests a return to more homogeneous transport, likely governed by trap-limited or ohmic conduction along the pre-formed CF. In contrast, during the switch from LRS to HRS (reset operation) under negative polarization, such an abrupt current change is not observed. This asymmetry between set and reset suggests that filament rupture proceeds via a kinetically slower mechanism, possibly involving gradual spatial redistribution within the switching CuO layer.

### 3.3. Preferential CF Formation at Grain Boundaries

**Figure 6:**
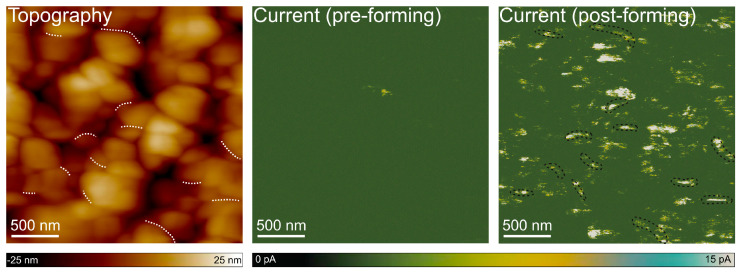
Topography and current maps acquired by C-AFM at the same location. The topography and the pre-forming current map were recorded simultaneously during the initial scan, showing negligible current flow. The post-forming current map was obtained in a subsequent scan and reveals localized high-conductivity spots, indicative of conductive filament formation. To highlight the correlation between surface morphology and conductivity, selected grain boundaries are marked with white dashed lines on the topography map, while corresponding regions exhibiting increased current on the post-forming current map are outlined with black contours.

To further investigate whether the RS observed in CuO-based memristor structures proceeds via the formation of localized conductive paths, C-AFM was employed to map the evolution of nanoscale current distribution across the structure surface before and after forming. This technique integrates topographical imaging with high-resolution current sensing. Therefore, it enables a direct correlation between morphological features and local conductivity. Again, to make the mapping possible, measurements were performed directly on the CuO layer and not on the electrical contact (Au). The force setpoint was kept constant during all scans to ensure repeatable electrical contact conditions. This value was carefully selected to minimize mechanical wear of the probe, especially the degradation of its conductive coating, thereby limiting its potential impact on the measurement accuracy both prior to and following the forming process. In these measurements, a PtIr-coated conductive top served as a top electrode, while the bottom contact was biased, allowing current mapping at a fixed read voltage of +1 V. A predefined 500 × 500 nm^2^ area was selectively subjected to localized forming by performing sequential I-V sweeps (0 to +6 V) at 400 discrete locations arranged in a 20 × 20 point grid with 25 nm distance between neighboring probing sites. The obtained current maps are shown in Figure 6.

Before the forming process, the current map exhibited a negligible signal. The root mean square (RMS) current signal recorded across the map was approximately 0.2, which is close to the setup detection limit, confirming the initially insulating nature of the CuO layer. In contrast, the post-forming scan revealed well-defined current hotspots that emerged selectively within the investigated area, and the RMS of the current signal increased to 7.8 pA. These relatively high-conductivity regions were spatially confined and distinctly absent in the surrounding non-switched zones, indicating a local, voltage-induced change in resistance. In this context, it is essential to underline that point-like changes in conductivity do not align with the interface trapping RS mechanism, which typically results in more uniform modifications across a broader region. Instead, the observed discrete and heterogeneously distributed high-current spots are characteristic of a filamentary switching mechanism, where the CFs form within the oxide layer.

The comparison between the topography (Figure 6) and the corresponding current maps reveals that these conductive spots do not coincide with the highest features in the morphology. Instead, they appear preferentially at or near grain boundaries. This spatial correlation supports the hypothesis that grain boundaries likely serve as energetically favorable sites for initiating and stabilizing conductive filaments. The enhanced local electric fields arising from structural irregularities at grain boundaries may lower the effective forming voltage threshold, further promoting the nucleation of CF at these interfaces. Interestingly, in the presented post-forming current map, some conductive features appear to be located within grain interiors. These may reflect sub-grain defects not visible in topography or slight variations in strain or local crystallography.

### 3.4. Post-Switching Microstructure Characterization via TEM and EDX

To provide direct evidence supporting the filamentary resistance switching mechanism, S/TEM was employed to investigate cross-sectional lamellae from devices previously subjected to switching cycles. Since all the earlier discussed results consistently pointed toward a CF-based mechanism, our goal was to identify unambiguous nanoscale morphological and compositional features associated with CF formation and rupture.

S/TEM techniques are well suited for this task, as they provide high-resolution imaging of nanoscale inhomogeneities and contrast variations, which may correspond to CF embedded within the CuO active layer. Nine cross-sectional lamellae were prepared using FIB from devices that had undergone forming and switching by C-AFM scanning within a several-nanometer-thick gold contact, targeting regions preserved in either the HRS or LRS prior to FIB milling.

Due to filament formation’s stochastic and localized nature, detecting CFs in randomly sampled lamellae is inherently challenging. Nonetheless, we identified several regions showing distinctive nanoscale features that are likely consistent with CF-related modifications. These include localized contrast variations within the CuO active layer and elemental segregation, suggesting the metallic species migration.

**Figure 7:**
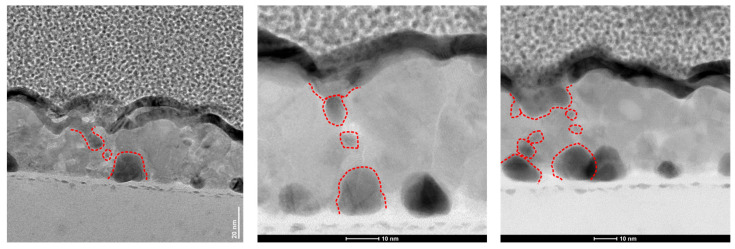
Cross-sectional TEM and BF-STEM images of the CuO-based device showing ruptured conductive filaments within 3 different areas of the switching layer. Areas marked in red indicate local contrast variations.

Figure 7 presents representative S/TEM images of the device in the HRS, revealing a set of contrast-modulated regions within the CuO layer. At the bottom interface of the active layer, Au-nanoseeds with diameters of approximately 10–15 nm are observed. Closer to the top electrode, some elongated dark regions appear, accompanied by smaller (~10 nm or below) features dispersed between these two zones. These morphological signatures are interpreted as agglomerates or clusters of gold atoms, which may reflect localized filament initiation, partial dissolution, or electromigration effects during RS cycles.

**Figure A2:**
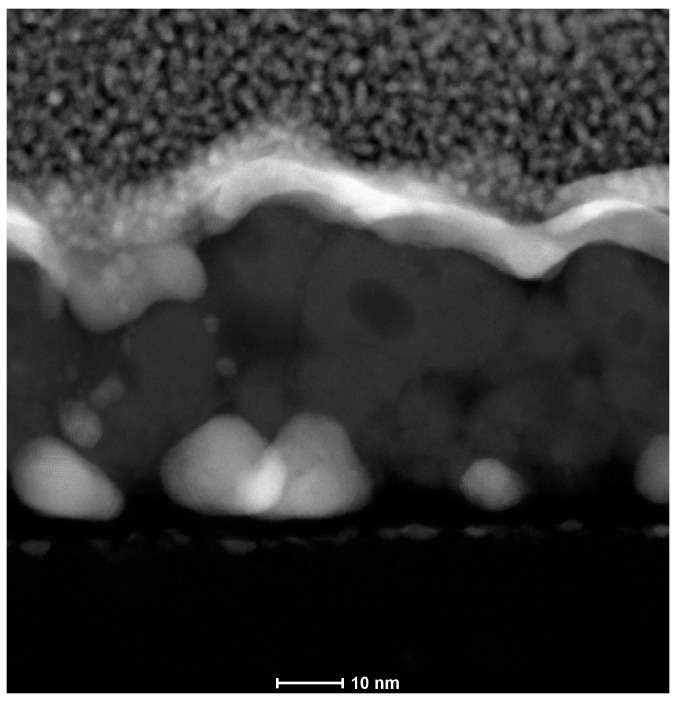
HAADF-STEM image of CuO-based memristor structure showing Au nanofeatures (bright regions) located along grain boundaries. The image corresponds to the region shown in Figure 7.

Crucially, these dark features appear preferentially aligned along grain boundaries, as seen more clearly in the last TEM image included in Figure 7 (especially in the HAADF-STEM image shown in the Appendix on Figure A2). This suggests that gain boundaries may act similarly to low-energy pathways for CF formation and rupture, which is consistent with the literature [37]. The observed interplay between Au-nanoseeds, grain boundaries, and localized variations in contrast supports the interpretation that resistive switching in these CuO-based memristive devices proceeds via a filamentary mechanism.

Since all conclusions up to this point were based solely on contrast variations in S/TEM images, complementary EDS analysis was conducted to determine the chemical composition of the observed filament-like structures unambiguously.

**Figure 8:**
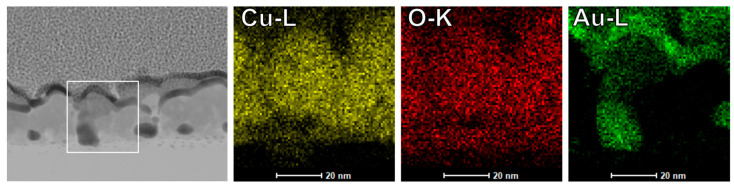
STEM image of the analyzed region (**left**) with the area of elemental mapping marked in white. Next, the corresponding elemental distribution maps for copper (Cu L-line), oxygen (O K-line), and gold (Au L-line) are shown.

Figure 8 presents a STEM image of the region under investigation, accompanied by the elemental distribution maps for oxygen, copper, and gold. The selected area was chosen from the same lamella discussed above, but from a different region that exhibits similar variations in contrast to those shown in Figure 7.

The distribution maps of oxygen and copper within the analyzed region appear uniform, indicating no significant structural or compositional inhomogeneities in the CuO layer. In contrast, the gold distribution map reveals a highly localized signal that spatially correlates well with the dark-contrast features related to CF that are visible in the STEM image. This spatial overlap confirms the presence of gold at these sites, thereby supporting the interpretation of CF formation via gold migration.

Moreover, based on earlier C-AFM experiments performed directly on CuO films (i.e., without the top Au contact), where RS was still observed, it can be hypothesized that the formation of CF proceeds from the bottom Au-nanoseeds toward the top electrode. However, unambiguous confirmation of the CF growth direction and dynamics would require in situ TEM analysis during device operation, which is planned in the future.

## 4. Discussion

The RS switching behavior observed in our gold-nucleated CuO-film-based memristive devices can be interpreted within the framework of the filamentary conduction mechanism, a widely recognized phenomenon in oxide-based memristors. In particular, two dominant models have been extensively described: the valence change mechanism (VCM) for Oxide-RAM (OxRAM) and the electrochemical mechanism (ECM), which underlies Conductive-Bridge RAM (CBRAM) [38]. In the VCM mechanism, RS is driven by the migration of oxygen anions or the redistribution of oxygen vacancies within the oxide active layer under an applied electric field. This leads to the formation of CF composed of oxygen-deficient regions. In contrast, the ECM mechanism observed in CBRAM devices involves the migration of metal cations from an active electrode to the dielectric layer, where they undergo redox reactions and form metallic filaments bridging the electrodes.

While VCM or hybrid VCM/ECM-type RS involving oxygen or copper vacancies has been reported for p-type CuO in other studies [21,39,40,41], our observations suggest a distinct and less common pure ECM-type filamentary mechanism in our memristive systems. This difference from commonly reported behavior is likely attributed to the unique growth technique employed in our work, which necessitates using Au-nanoseeds to promote the hydrothermal growth of CuO on the substrate surface. Unlike other studies that rely on physical or chemical deposition methods and do not require nucleating layers, our solution-based approach imposes different boundary conditions at the bottom electrode–oxide interface, which critically affect both the CF growth and the species involved in their formation.

The experimental evidence supporting this hypothesis is multifaceted. First, the I-V characteristics presented on a double-logarithmic scale show an abrupt HRS-to-LRS switch and a large ON/OFF ratio, which is consistent with the filamentary mechanism. Second, C-AFM maps obtained after forming show highly localized areas of increased current, suggestive of the formation of nanoscale conduction channels upon electrical stress. Although C-AFM provides only surface-sensitive information and cannot directly resolve the vertical geometry of the CFs, the observed current localization near grain boundaries strongly supports the filamentary mechanism.

Third, and most importantly, S/TEM and EDX mapping reveal the presence of non-uniform Au distributions within the CuO switching layer. These Au-rich regions, extending from gold nanoseeds to the top gold electrode, indicate directional mass transport of Au. In contrast, the spatial distribution of Cu and O remains uniform, proving no evidence of Cu migration or compositional changes in the CuO layer. The evidence presented strongly excludes the valence change mechanism and suggests that gold (Au) serves as the mobile species.

The observation that the filaments form preferentially at grain boundaries supports the theory that such microstructural features offer energetically favorable sites for ion transport and redox reactions typical for the ECM mechanism. Therefore, the grain boundaries create optimal conditions for ion migration and, consequently, for the formation of CFs in these regions, thereby explaining the confined nature of the C-AFM high-current spots. This switching scenario implies that the CuO film, rather than participating directly in the redox reactions, acts primarily as a passive medium facilitating the ion transport. The involvement of a noble metal such as Au in ECM-like processes is uncommon because of its high redox potential. However, such behavior becomes possible under nanoscale confinement and high electric fields and has been previously reported for HfO_2_ or MoS_2_-based systems [42,43,44]. Our findings extend this concept to CuO, highlighting the importance of the electrode–oxide interface and likely local field enhancement near nanoscale features.

## 5. Conclusions

In this study, we employed a complementary set of advanced characterization techniques to confirm the filamentary resistive switching mechanism in our gold-nucleated CuO film-based memristive devices. Electrical measurements and nanoscale imaging together contributed to the understanding of RS behavior. A key result is provided by S/TEM and EDS analysis, which not only directly confirmed the formation of CF but also revealed that these CF consist of Au nanoclusters. This observation allowed us to identify the RS mechanism as an ECM-type filamentary process based on the formation of metallic bridges between the bottom electrode, specifically, the Au-nanoseeds used as nucleation sites for CuO growth on the substrate, and the top electrode.

Although this direct evidence is compelling, definitive proof of the filament formation dynamics requires in situ TEM observations under device operation, which remains a goal for future work. Based on the available data, we propose that the CF forms from Au-nanoseeds towards the top electrode, through grain boundaries. This is further supported by the RS observed when the top Au electrode was replaced by a PtIr-coated conductive AFM probe, indicating that the only source of Au in such a device was the nanoseeds.

This novel insight opens a promising research direction focused on the influence of the nucleating seed material and its parameters on the resistive switching phenomenon in CuO-based memristive devices. Although the present study provides strong evidence for Au migration as the dominant factor in resistive switching in our structures, the detailed chemical reactions remain to be fully elucidated. Future investigations will comprehensively understand these fundamental aspects, particularly ion transport and filament formation dynamics, to optimize device performance and reliability.

## 6. Patents

The described research concerns the following patented by the Patent Office of the Republic of Poland solutions: “Hydrothermal method of producing a CuO layer on a substrate” (Pat.241026) and “A method of producing a memory structure and the memory structure produced by this method” (Pat.246583)

## Data Availability

The original contributions presented in the study are included in the article; further inquiries can be directed to the corresponding authors.

## Appendix A

Figure A1 below shows SEM images of identically nucleated (in a single sputtering process) samples taken from the reaction vessel every 30 s during the growth process. The obtained results indicate the mechanism of layer formation. Nanocrystals of CuO form initially on the nanoseeds. These nanocrystals then act as nucleating sites for further growth. Such grown CuO structures agglomerate, leading to the grain formation. As the process continues, these grains grow and densify, resulting in the formation of a continuous layer on the substrate. It is worth noting that when the hydrothermal process was conducted on unseeded substrates, CuO formed exclusively as powders suspended in the solution volume.

To better visualize the spatial distribution of Au nanoclusters with the grain boundaries within the polycrystalline CuO layer, in Figure A1, we present a HAADF-STEM image of the region shown in Figure 7. This signal highlights more clearly that the Au-rich nanofeatures are well aligned along the grain boundaries, reinforcing our conclusion about the preferential filament formation sites.
